# Cobalamin coenzyme forms are not likely to be superior to cyano- and hydroxyl-cobalamin in prevention or treatment of cobalamin deficiency

**DOI:** 10.1002/mnfr.201500019

**Published:** 2015-05-12

**Authors:** Rima Obeid, Sergey N Fedosov, Ebba Nexo

**Affiliations:** 1 Aarhus Institute of Advanced Studies,, University of AarhusAarhus, Denmark; 2 Department of Clinical Chemistry, University Hospital of the SaarlandHomburg, Germany; 3 Department of Clinical Biochemistry, Aarhus University HospitalAarhus C, Denmark; 4 Department of Molecular Biology and Genetics, Aarhus UniversityAarhus C, Denmark

**Keywords:** Adenosylcobalamin, Coenzyme, Cyanocobalamin, Deficiency, Hydroxylcobalamin, Methylcobalamin

## Abstract

Methylcobalamin (MeCbl) and adenosylcobalamin (AdoCbl) are coenzymes for methionine synthase and methylmalonyl-CoA mutase, respectively. Hydroxylcobalamin (HOCbl) and cyanocobalamin (CNCbl) are frequently used for supplementation. MeCbl and AdoCbl have recently emerged as alternative forms in supplements. In the light of metabolic transformation of Cbl into its cofactor forms, this review discusses current evidence on efficacy and utility of different Cbl forms in preventing or treating Cbl deficiency. Cbl-transporting proteins bind and mediate the uptake of all aforementioned forms of Cbl. After internalization and lysosomal release, Cbl binds to the cytosolic chaperon MMACHC that is responsible for (i) flavin-dependent decyanation of [CN-Co^3+^]Cbl to [Co^2+^]Cbl; (ii) glutathione-dependent dealkylation of MeCbl and AdoCbl to [Co^2+/1+^]Cbl; and (iii) glutathione-dependent decyanation of CNCbl or reduction of HOCbl under anaerobic conditions. MMACHC shows a broad specificity for Cbl forms and supplies the Cbl^2+^ intermediate for synthesis of MeCbl and AdoCbl. Cobalamin chemistry, physiology, and biochemistry suggest that MeCbl and AdoCbl follow the same route of intracellular processing as CNCbl does. We conclude that supplementing MeCbl or AdoCbl is unlikely to be advantageous compared to CNCbl. On the other hand, there are obvious advantages of high parenteral doses (1–2 mg) of HOCbl in treating inborn errors of Cbl metabolism.

## 1 Introduction

Vitamin B12 (cyanocobalamin, CNCbl) was discovered in the first half of the 20th century and identified as the anti-pernicious anemia factor. The two coenzyme forms, 5′-deoxy-5′-adenosylcobalamin (AdoCbl) and methylcobalamin (MeCbl), were discovered later on [1,2]. In mammalian cells, MeCbl is a cofactor for the cytosolic methionine synthase, whereas AdoCbl is a cofactor for the mitochondrial methylmalonyl-CoA (MM-CoA) mutase. Hydroxycobalamin (HOCbl) is an abundant and physiologically relevant intermediate form. Exposure of aerated aqueous solutions of MeCbl and AdoCbl to ambient light causes formation of HOCbl *in vitro*. Cobalamin (Cbl) is necessary for synthesis of DNA bases, transfer of methyl group [i.e. regeneration of S-adenosylmethionine (SAM)], as well as metabolism of branched chain amino acids and fatty acids with an odd number of carbon atoms.

There are a variety of Cbl forms, which share the core structure of Cbl but contain different upper ligands. CNCbl is a stable and inexpensive synthetic form commonly used for food fortification and oral or parenteral supplements. Also the physiological forms of cobalamin (HOCbl, AdoCbl, and MeCbl) are available as supplements with different routes of administrations. When supplemented, CNCbl needs to be converted into MeCbl and AdoCbl in order to exert its anticipated biological effect on the cell. The concept of replacing CNCbl/HOCbl with the coenzyme forms as ready-to-use sources of the cofactors has recently emerged [3,4]. Supplementation of MeCbl and AdoCbl is postulated to be more effective than that of CNCbl/HOCbl [3,4]. Direct providing of the active Cbl forms to deficient people is attractive, but two questions remain debatable: (i) can MeCbl or AdoCbl reach their final destination in the cell in the original unmodified forms to function as the ready coenzymes? (ii) Is the level of evidence sufficient to recommend this rather expensive concept as an alternative to CNCbl/HOCbl? This review discusses the available evidence on the efficacy and utility of different Cbl forms in preventing or treating Cbl deficiency in the light of Cbl chemistry, transport, and intracellular metabolism.

## 2 Cobalamin deficiency

The recommended dietary allowance (RDA) for Cbl for adults as set by the US Institute of Medicine is 2.4 μg/day [5]. A common cause of Cbl deficiency is low dietary intake (i.e. vegan diet and poor nutrition) [6]. This condition can be prevented by including animal source foods in the diet or providing Cbl through oral supplementation (i.e. up to 10 μg/day). However, a sufficient intake of the vitamin does not always ensure an optimal Cbl status. Mild to moderate Cbl deficiency is common in industrialized countries [7,8] despite that a typical western omnivorous diet provides approximately 5–7 μg Cbl/day. This may be explained by an age-related decrease in the ability to release Cbl from the food [9,10] or by an impaired intestinal absorption of Cbl. Pernicious anemia is an autoimmune disease that leads to a deficient production of intrinsic factor (IF), the protein that facilitate Cbl absorption. The latter condition can cause severe Cbl deficiency even at adequate dietary intake of the vitamin. Cbl deficiency in individuals with food Cbl malabsorption can be prevented by providing oral Cbl supplements containing < 0.5 mg/day, while patients with pernicious anemia need pharmacological doses of Cbl, either orally (≥ 0.5 mg/day) or parenteral in doses of ≥ 1 mg given at regular intervals.

## 3 Cobalamin chemistry

**Figure 1:**
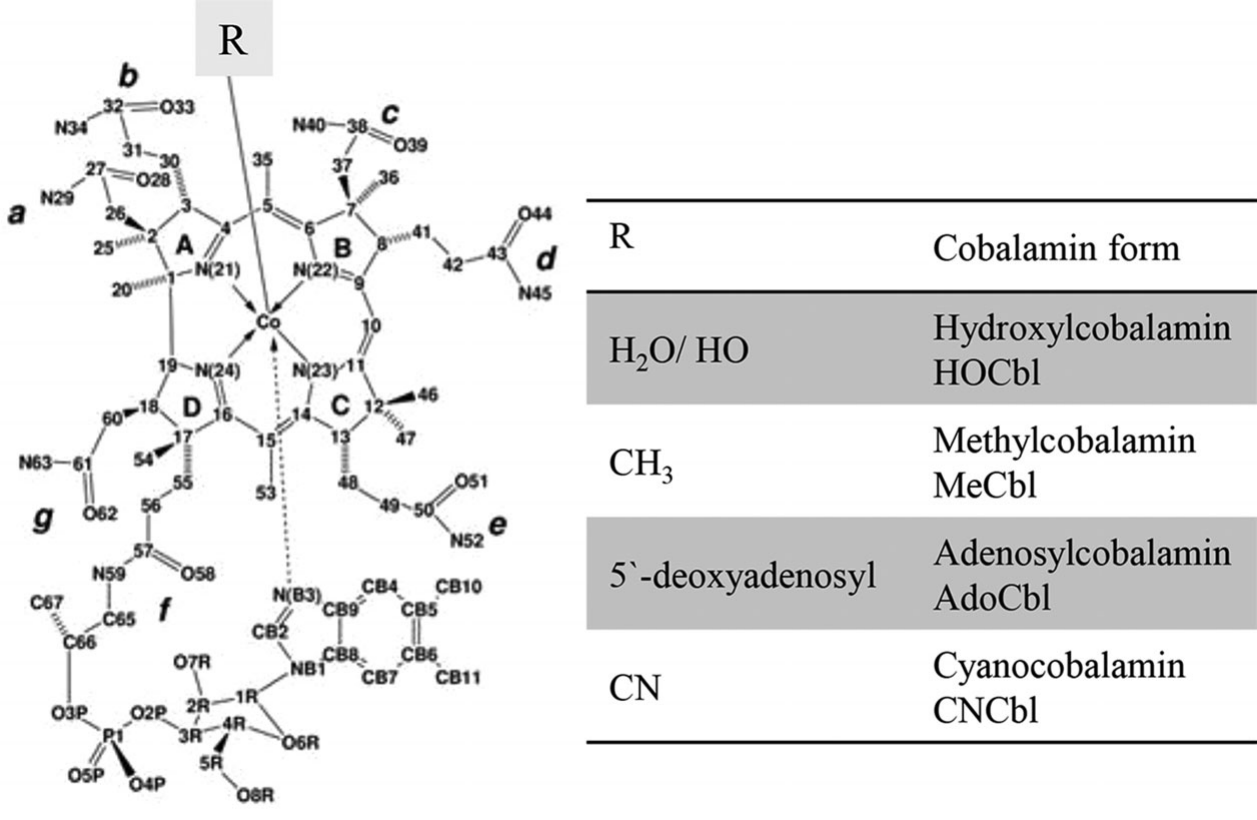
Vitamin B12 chemical structure and forms. R is bound to the upper (β-face).

Cbl is a collective name for structurally related compounds that contain a central cobalt (Co) ion coordinated to four equatorial nitrogen atoms donated by the tetrapyrrolic corrin ring. The Cbl structure includes two axial positions, where different ligands can coordinate to the Co ion (usually Co^3+^). The ligand on the upper surface of the ring (β-face) may be cyanide (CN^−^), water (H_2_O or HO depending on pH of the surroundings), or an alkyl-group [i.e. methyl (Me) or 5′-deoxyadenosyl (Ado)], or other ligands (Fig. 1). The Me group in MeCbl is among the strongest coordination ligands, whereas water of HOCbl belongs to the weakest coordination ligands. Under *in vitro* conditions at room temperature, the Co–carbon bond of dissolved MeCbl and AdoCbl does not dissociate, but it breaks under light exposure leading to the formation of HOCbl plus formaldehyde (from MeCbl) and cyclic adenosine/adenosine aldehyde (from AdoCbl). *In vivo*, the Co–carbon bond of MeCbl and AdoCbl is cleaved and restored under enzymatic catalysis (see below).

CNCbl, MeCbl, and AdoCbl are neutral molecules at physiological pH. HOCbl occurs as either charged molecule (H_2_OCbl^+^) at neutral and slightly acidic pH or uncharged molecule (HOCbl^0^) at alkaline pH. The nature of Cbl-form and its enzyme determine the mechanism of dissociation of the Co–carbon bound. Methyltransferases cleave MeCbl heterolytically, where the methyl-group (formally CH_3_^+^) is transferred from ^–^Cbl (i.e. [Co^1+^]Cbl with two “excessive” electrons) to a negatively charged acceptor like homocysteine. This transfer is a consequence of a direct attack of e.g. biological thiols on Me of MeCbl. MM-CoA mutase cleaves AdoCbl homolytically thus generating two radicals Ado**^·^** and **^·^**Cbl (i.e. [Co^2+^]Cbl) [11]. At the end of the catalytic cycle, the cofactors are restored to their original forms MeCbl and AdoCbl (i.e. Me-[Co^3+^]Cbl and Ado-[Co^3+^]Cbl). The Co-ion in the center of Cbl can exist in three oxidation states (Co^3+^, Co^2+^, and Co^1+^), which appear in a strict sequence under the aforementioned catalytic transformations.

The nucleotide base, 5,6-dimethylbenzimidazole (DMB), constitutes the ligand on the lower surface of the corrin ring (α-face) in cobalamins. The oxidation state of Cbl affects the association of the upper and lower ligands of the corrin ring. [Co^1+^]Cbl has no axial ligands coordinated to the Co-ion, whereas [Co^2+^]Cbl and [Co^3+^]Cbl have one and two axial positions occupied by DMB and DMB + upper ligand, respectively. In general, [Co^3+^]Cbl and [Co^2+^]Cbl exist in solution in their “base-on” forms, meaning that DMB is coordinated at the α-face of the corrin ring. The Co-N coordination bond between cobalt and DMB is, however, not strong, and the nucleotide base can dissociate depending on pH, temperature, protein environment, and oxidation state of the Co-ion. Cbl-configuration with the dissociated base is called “base-off”. Cobalamin transporting proteins in mammals [haptocorrin (HC), IF, transcobalamin (TC)] recognize and bind the “base-on” cobalamins. In contrast, Cbl-dependent MM-CoA mutase, methionine synthase, and chaperon MMACHC bind Cbl in a “base-off” form. The switch between “base-on” and “base-off” configurations modifies the reactivity of the Co-associated upper ligand and enables the changes in redox-chemistry needed for the enzymatic activity [12].

## 4 Cellular trafficking and metabolism of cobalamin

**Figure 2:**
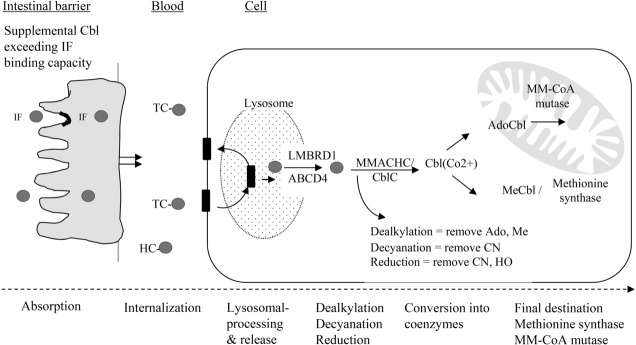
Trafficking and assimilation of all forms of Cbl into mammalian cells is likely to follow the same steps: crossing the intestinal barrier, cellular internalization, lysosomal release, dealkylation, decyanation or reduction, forming the coenzymes and then attachment to the corresponding enzymes. Ado; adenosyl, CN; cyano-, HC; haptocorrin, HO; hydroxyl, IF; intrinsic factor, LMBRD1 and ABCD4; are lysosomal membrane exporters, Me; methyl, MMACHC; methylmalonic aciduria and homocystinuria type C protein, MM-CoA: methylmalonyl-CoA, TC; transcobalamin.

Cbl can be provided by the diet as protein bound-Cbl, as free crystalline Cbl in fortified foods, or by supplementation through different routes (oral, parenteral, and nasal). After oral administration of physiological (up to 10 μg) or therapeutic doses (≥ 0.5 mg), all Cbl forms must first cross the intestinal barrier and second cross the cell membrane before they encounter the Cbl-dependent enzymes (Fig. 2). Most work concerning uptake of Cbl has been conducted using CNCbl. Theoretically, if Cbl-coenzymes are effectively taken up and remain unchanged under the transport and intracellular processing, they can be regarded as ready-to-use coenzymes. In contrast, a potential superior role for Cbl coenzymes appears to be less plausible if all Cbl forms follow the common route of reduction-oxidation in the cell.

### 4.1 Cobalamin trafficking and internalization into the cells

Dietary Cbl reaches the circulation by an active receptor-mediated intestinal absorption. Delivery of Cbl from food or supplements into the cell relies on successive proteins of extracellular transport, in humans these are HC, IF, and TC. Releasing of food-bound Cbl in the stomach requires acid and pepsin. Thereafter, Cbl is trapped by HC (originating from saliva) and travels with HC to the duodenum, where trypsin and other intestinal proteases cleave HC, thereby allowing transfer of Cbl from HC to IF, whereas inactive corrinoids, Cbl–analogues, remain bound to HC fragments. IF is a specific Cbl binding protein produced by the parietal cells and is able to bind Cbl at neutral pH. In the terminal ileum, IF-Cbl is internalized by cubillin that is a part of the receptor cubam present on the luminal site of enterocytes. In the enterocyte, cubam is recycled, IF is degraded and Cbl is delivered into the bloodstream, most likely supported by the intestinal basolateral transport protein MRP1 [13]. The IF-mediated intestinal absorption is limited to around 1.5 μg per meal because of the limited pool of cubam [14]. The newly absorbed Cbl appears in blood bound to TC that is responsible for delivering Cbl into the cells. TC-Cbl is rapidly cleared from the circulation by cellular uptake mediated by the TC-receptor (CD320) or by filtration in the kidney followed by re-uptake in the proximal tubule.

All three Cbl-transporting proteins bind Cbl with high affinities. IF followed by TC show the highest specificity for Cbl [15–17]. The binding of Cbl to the transporters is fast, exceptionally tight, and highly selective for the Cbl “base-on” configuration [18–21]. In contrast to Cbl forms mentioned above, Cbl-analogues have low affinity for IF and TC, but they bind to HC that is thought to act as a scavenger for biologically inactive analogues [22]. The transporting proteins form a sandwich-like compact structure where two domains of the protein wrap the Cbl molecule, thus protecting up to 95% of its surface. Both human and bovine TC provide additional protection of the chemically vulnerable upper face of HOCbl. This is achieved by coordination of a His-residue from TC and displacement of water from the β-site of Cbl. On the contrary, IF and HC lack this feature.

Cbl-transporters have a significant tolerance to Cbl forms with different upper groups (Me, Ado, HO, or CN) [16,23]. The binding constants of CNCbl or HOCbl (both containing small β-groups) with the transporters are comparable to that of AdoCbl (containing a relatively large β-group). HOCbl, AdoCbl, and MeCbl bind to IF and induce shrinkage of the protein (measured as Stokes radius) similar to that caused by CNCbl [24]. Although the solvent-accessible surface of the upper ligand corresponds to only 7% in TC-HOCbl complex (more compact structure), 17% in TC-CNCbl complex, and 19% in the IF-CNCbl complex [15,25], none of the upper *β*-ligands tested so far plays a critical role in the transporter recognition of different Cbl forms [26].

### 4.2 Transport of Cbl within the cell and formation of Ado- and MeCbl

CD320-TC-Cbl complex is internalized into the lysosomes whereupon CD320 is recycled to the cell surface, while Cbl is released after TC-proteolysis [27]. Subsequently, Cbl is released from the lysosomes into the cytosol by the membrane bound transport proteins, LMBD1 and ABCD4 [28,29]. The lysosomal release of Cbl is impaired *in cblF* and *cblJ*, disorders caused by a loss of function in LMBD1 or ABCD4 proteins [28,30,31]. Cultured fibroblasts from affected patients accumulate CNCbl in lysosomes and are unable to produce AdoCbl or MeCbl from CNCbl added to the medium. Consequently, the activities of the mitochondrial mutase and cytosolic methionine synthase are reduced in cblF and cblJ fibroblasts. The neurological symptoms in affected patients mostly persist under treatment with CNCbl, but the Cbl-dependent enzymes are activated by administration of therapeutic doses of HOCbl (≥ 1 mg) [32,33], suggesting the ability of HOCbl (but not CNCbl) to bypass the classical processing route. MeCbl was not tested in this context. *In vitro* lysosomal accumulation of CN[^57^Co]Cbl can be induced by using pH-dependent or independent lysosomal proteolysis inhibitors [34] that reduce cytosolic and mitochondrial Cbl-contents and decrease activity of MM-CoA mutase [34]. Therefore, lysosomal processing is mandatory for subsequent production of Cbl cofactors under physiological conditions (no therapeutic doses tested).

**Figure 3:**
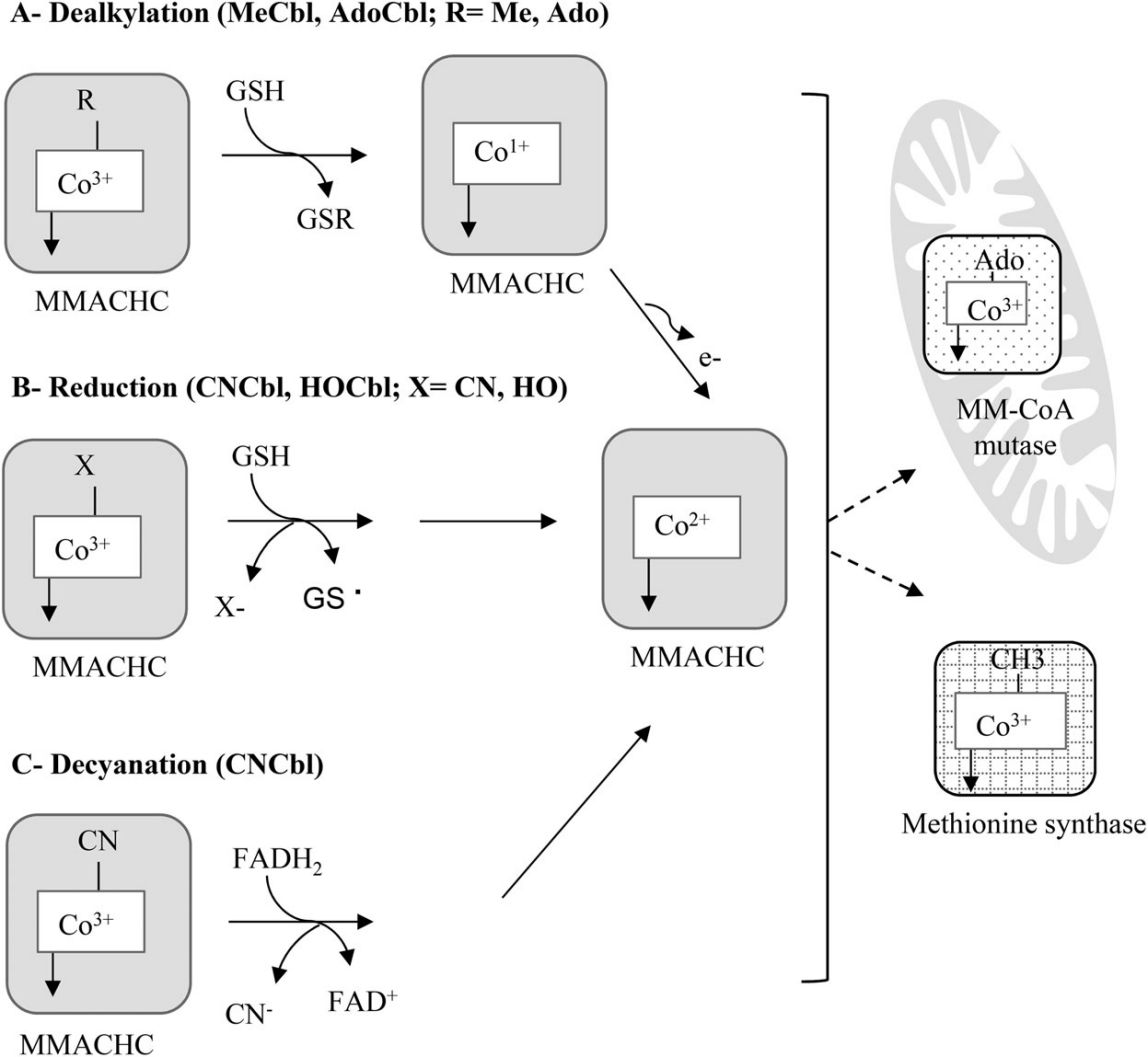
Human MMACHC in the cytosol mediates the removal of the upper ligand in all Cbl forms (MeCbl, AdoCbl, HOCbl, and CNCbl). Dealkylation removes Me or Ado and requires glutathione S-transferase; decyanation of CNCbl requires a flavin reductase system (FAD), and a reduction in the presence of GSH leads to decyanation under anaerobic conditions or removal of HO group. Under all circumstances, a Co^+2^ intermediate is produced. AdoCbl; adenosylcobalamin, CNCbl; cyanocobalamin, FAD; flavin adenine dinucleotide, HOCbl; hydroxylcobalamin, GSH; glutathione, MeCbl; methylcobalamin, MMACHC; methylmalonic aciduria and homocystinuria type C protein, MM-CoA: methylmalonyl-CoA.

Once Cbl is released to the cytosol, the cytosolic chaperon, methylmalonic aciduria, and homocystinuria type C protein (MMACHC, called also CblC) removes the upper ligand of Cbl via decyanation, dealkylation, or reduction and prepares Cbl for being methylated or adenosylated [35–37]. These conversions are assisted by methionine synthase reductase (MSR) plus flavins and NADPH, or require reduced glutathione (GSH). MMACHC is a cytosolic Cbl-trafficking protein that exhibits a broad specificity for potential different incoming Cbl provided by supplementation (i.e. CN-, Me-, Ado-, OH-) [38]. Cbl-binding to MMACHC is associated with a conformational transition from the “base-on” to the “base-off” configuration. Human MMACHC conducts decyanation and dealkylation by two different mechanisms [35,39]. A flavin-MSR-dependent reduction removes the upper CN-group or reduces HOCbl. Additionally, dealkylation (removal of Me or Ado) is mediated by a GSH-dependent reduction that under anaerobic conditions also removes HO- and CN- groups [35] (Fig. 3).

Defects in MMACHC (*cblC* gene) lead to inability to synthesize MeCbl and AdoCbl from supplemental CNCbl [40], but patients show response to 1–2 mg of HOCbl [41] that enhances both methionine synthase and MM-CoA mutase activities. HOCbl has also been shown to induce methionine synthase activity *in vitro* [42]. The exposed nature of the Co-ion in HOCbl possibly facilitates its nonspecific chemical reduction to [Co^2+^]Cbl, which afterward can be converted to the coenzymes. One of the potential mechanisms might include glutathione-dependent reduction 2·GS^−^ + HOCbl^+^ → GSSG + [Co^1+^]Cbl^−^ followed by gradual oxidation [Co^1+^]Cbl^−^ → [Co^2+^]Cbl^0^ → [Co^3+^]Cbl^+^ with a necessary intermediate [Co^2+^] generated. A low efficient bypass of the MMACHC is unlikely in individuals with a normally functioning MMACHC and/or MSR. The latter enzyme reduces free HOCbl to its [Co^2+^]-form in the presence of flavin and NADPH. The current evidence suggests that when supplemented, CNCbl, MeCbl, and AdoCbl are likely to follow the same path via MMACHC giving rise to [Co^2+^]Cbl. In contrast, if HOCbl can reach the cytosol after supplementation, the MSR-dependent enzymatic conversion or a spontaneous GSH reduction of HOCbl may partly rescue the cofactor function of this Cbl form.

Removing the upper group of Cbl is essential for the biological functions of the vitamin. Bovine endothelial cells are able to produce AdoCbl and MeCbl by dealkylation of [^57^Co]-labeled xenobiotic alkylcobalamins with an increasing β-axial ligand alkyl chain length [39]. The isolated MMACHC catalyzes the rapid demethylation of MeCbl within few minutes *in vitro*. On the contrary, artificial arylcobalamins have stronger Co-C bonds that cannot be efficiently reduced by MMACHC [38]. All Cbl ligands susceptible to reduction by MMACHC possess the vitamin potential, whereas Cbl forms resistant to this step show the anti-vitamin effect (i.e. 4-Ethylphenylcobalamin) [43]. The removal of the upper ligand of Cbl by MMACHC is followed by processing via MMADHC protein (cblD gene product) that acts as a branch point and delivers the reduced [Co^2+^]Cbl to either cytoplasm for formation of MeCbl or mitochondria for formation of AdoCbl [44,45].

Taken together, the passage through LMBD1 and ABCD4 steps is obligatory in order to release Cbl from the lysosomes under physiological conditions. Dealkylation or decyanation via MMACHC is obligatory for further synthesis of Cbl coenzymes [39]. It is currently not known whether Cbl (i.e. in the coenzyme form) can bypass this pathway and can reach the enzymes if administered at therapeutic doses. This assumption is unlikely because otherwise, CNCbl would be expected to inhibit the Cbl-dependent enzymes, which is not the case under treatment with high doses of CNCbl (> 1mg). Considering a similar intracellular fate of CNCbl, MeCbl, and AdoCbl, we find it very unlikely that either MeCbl or AdoCbl is superior to CNCbl. Current evidence does not lend support to the hypothesis that MeCbl or AdoCbl may bypass this general route. Lessons learned from genetic defects of Cbl suggest that compared to other Cbl forms, HOCbl may prove superior [39] as is further elaborated below.

### 4.3 Cobalamin forms and activation of Cbl-dependent enzymes

The upper ligand of Cbl has a major role in activation of the Cbl-dependent enzymes. Methionine synthase and methylmalonyl-CoA mutase (MUT) require MeCbl and AdoCbl, respectively. HOCbl appears to partly induce the activity of both enzymes, thus explaining the partial effect of high doses of HOCbl (i.e. 0.5–2 mg orally or i.m.) in treating patients with defects in Cbl metabolism [46,47].

The activity of methionine synthase in cell extracts is induced by HOCbl and MeCbl and to a lower degree by CNCbl [48]. HOCbl shows faster induction of methionine synthase *in vitro* compared to CNCbl and MeCbl [42]. However, all three forms show similar methionine production (as a marker for methionine synthase activity) when the incubation is continued over 60 min [42]. The quick effect of HOCbl is probably caused by its susceptibility to reduction by GSH or/and MSR + NADPH after entering the cell [35,49,50].

## 5 Evidence on potential effectiveness of different Cbl forms

CNCbl and HOCbl are employed for prevention or treatment of Cbl deficiency in Europe and the US. In Asia, MeCbl is the popular form, whereas AdoCbl is available but less popular. Considering clinical trials as a proof of therapeutic effectiveness, there is no sufficient evidence to recommend one of the forms as a better option for prevention or treatment. Almost all published clinical studies using CNCbl, HOCbl, or MeCbl are non-controlled open label trials that used therapeutic doses of Cbl (between 0.5 and 2.5 mg) (Tables 1 and 2, Supporting Data File). AdoCbl has not been studied enough.

**Table 1:** Unresolved issues regarding the biological effects of Cbl forms

Level of effect	Unresolved issues
Intestinal absorption after oral supplementation	• Is the interaction of IF-Cbl with cubam and the internalization process influenced by the Cbl-form bound to IF?
	• Is the percentage of Cbl (i.e. 1–2% of the oral dose) that can pass into the circulation by passive diffusion different between the Cbl forms when high oral Cbl doses (0.1–1 mg) are used? (after saturating IF-mediated uptake)
	• The pharmacokinetic of AdoCbl has not been sufficiently studied to judge its absorption, conversion or potential benefit.
	• Are there any differences in the absorption, bioavailability and intracellular processing when low doses of the different forms are utilized (≤ 10 μg)?
Cell internalization via CD320	• Can CD320 equally recognize and internalize the different form of Cbl?
	• Can therapeutic doses of Cbl (i.e. 1 mg i.m.) enter the cell without the CD320 receptor? Can they bypass the lysosomal pathway, or MMACHC reduction?
Intracellular processing	• Are Cbl forms different in the speed of or the efficacy of their release from the lysosome, decyanation or dealkylation /or reduction?
	• Can Cbl forms leave the cell?
Blood and intracellular forms	• What is the effect of supplementing a certain Cbl form on the distribution of the other forms? Is this effect dose- and/or time-dependent?

Adams et al., investigated the absorption of labeled CNCbl, MeCbl, HOCbl, and AdoCbl in humans at a relatively low single oral dose [51]. After ingestion of 1 μg, the percentage of Cbl retained in the body ranged between 34% for AdoCbl and 56% for HOCbl [51]. At a dose of 5 μg, 13% of the ligand was retained after AdoCbl administration and 20% after CNCbl. Administration of 25 μg showed 6% retention for CNCbl and 8% for AdoCbl [51]. Available studies have some limitations and they do not support consistent clear differences between Cbl forms in term of absorption, tissue clearance, or clinical effects [52].

HOCbl binds to the transporters, shows uptake by the cells, and enhances activity of Cbl-dependent enzymes [53–55]. In clinical trials, HOCbl (400 μg i.m.) lowers methylmalonic acid and homocysteine [53]. HOCbl accounts for approximately 50% of the Cbl on TC in blood of non-supplemented individuals [56], probably highlighting the availability or tightness of TC-binding to this form. HOCbl (200 μg i.m.) shows a longer retention in plasma and a lower excretion in urine than an equivalent dose of CNCbl does [57,58]. However, a wide inter-individual variations in HOCbl retention have been reported [58], suggesting that physiological factors (i.e. renal function, degree of tissue depletion) may interfere with the response. HO[Co^57^]Cbl (∼17 ng/intracardial injection) shows higher uptake to the cytosol and the mitochondrial fractions in rats [54]. In cell culture models, HOCbl (1 ng/10^6^ cells) showed a more efficient conversion into coenzymes as compared to labeled CNCbl [57]. HOCbl is advantageous in case of treating genetic disorders of Cbl. Compared with CNCbl, the faster conversion of HOCbl to coenzymes and its extended effect in human [57] can be explained by a faster reduction of HOCbl and unspecific binding to proteins (i.e. albumin) via Co-NH_2_ or Co-His coordination. The “unspecific” binding to albumin increases the retention of HOCbl in plasma and constitutes a ready-to-use reservoir of the vitamin.

Using HOCbl is advantageous in two exceptional conditions. First, HOCbl is used to treat cyanide intoxication, because it coordinates CN^−^ with a very high strength and increases cyanide elimination via kidney filtration [59]. Second, supplementation of HOCbl is favored in some rare genetic defects of Cbl metabolism, when MeCbl and AdoCbl cannot be formed in the cell from CNCbl [60]. As outlined above, HOCbl appears to partly bypass the conventional route of Cbl processing via MMACHC necessary to induce the activity of methionine synthase and MM-CoA mutase [33]. Therefore, some advantages of HOCbl over CNCbl can be assumed based on its pharmacokinetic features and potential to circumvent MMACHC, particularly after parenteral application at therapeutic doses (1-2 mg). However, the low stability of HOCbl may make it less suited for oral supplementation.

Like HOCbl, CNCbl binds to Cbl-transporters and provides the anticipated effects on metabolic and clinical levels. Oral supplementation of low doses of CNCbl (3 × 9 μg/day) causes appearance of this form in blood, but also a significant increase of MeCbl-bound to TC [56], suggesting that a conversion to the cofactor MeCbl has taken place just after supplementation. CNCbl not bound to Cbl-binding proteins is rapidly cleared from the circulation via the kidney. When low doses of Cbl are injected (10 × 54 μg), the percentage of Cbl excreted in urine (14% of the sum of Cbl doses) was lower when compared to a larger dose of 10 × 1 mg (where 70% were excreted) [61].

MeCbl (25 mg/day for 10 days, then 25 mg/month i.v.) showed clinical benefit [62], lowered homocysteine (when 1 mg /day i.m. was applied for 3 weeks) [4,63] (Table 2, Supporting Data File) and specifically induced the activity of methyltransferase *in vitro* (compared to AdoCbl, OHCbl, or CNCbl) [64].

It has been an issue whether pharmacological doses of MeCbl can act as a nonspecific methyl donor or an effector of other methylation processes than the conversion of homocysteine to methionine. *In vitro*, methyl groups from MeCbl are incorporated into DNA via DNA-methyltransferase (from rat spleen) when SAM is absent [65]. In the presence of SAM (which is abundant in the physiological milieu), also AdoCbl and CNCbl increase DNA-methylation by methyltransferase, though MeCbl is the most efficient compound [65]. Thus, it appears that the role of MeCbl as a universal direct methyl donor *in vivo* is uncertain because SAM and GSH are abundant in the cell. MeCbl, not bound to the specific Cbl binding proteins, is likely to be freely filtered in the kidney, thus it is likely that free MeCbl and CNCbl will have similar turnover times in plasma. Taken together, HOCbl may be better for parenteral treatment. MeCbl and CNCbl show very similar biological effects, but CNCbl may be superior as oral supplementation because of its stability.

## 6 Conclusions and unresolved questions

At doses commonly used in treatment or prevention of Cbl deficiency, all Cbl forms appear to be absorbed, internalized by the cells and follow the intracellular metabolic pathway that converts all forms into the common intermediate [Co^2+^]Cbl that in its turn transfers into the functional coenzymes, MeCbl, and AdoCbl. MMACHC exhibits a broad specificity for different Cbl forms and removes the upper ligand of incoming cobalamins, thus MeCbl and AdoCbl coenzymes can be synthesized from all available Cbl forms. Available clinical studies are non-controlled trials and almost all of them show the beneficial biological effects independent of the form that has been used.

Currently, we do not have sufficient evidence to suggest that the benefits of using MeCbl or AdoCbl override that of using CNCbl or HOCbl in terms of bioavailability, biochemical effects, or clinical efficacy. There is uncertainty regarding the claimed superior role of Cbl coenzyme forms for prevention and treatment of Cbl deficiency (Table 1). However, HOCbl may be an advantageous precursor of the cofactors, particularly in the inherited disorders of metabolic Cbl processing. CNCbl is a more stable and inexpensive form that appears to be best suited for oral supplementation and parenteral treatment as well.

## Abbreviations

AdoCbl: 5′-adenosylcobalamin
Cbl: cobalamin
Co: cobalt
CNCbl: cyanocobalamin
d: day
DMB: 5,6-dimethylbenzimidazole
FAD: flavin adenine dinucleotide
GSH: glutathione
HC: haptocorrin
HOCbl: hydroxylcobalamin
Hcy: homocysteine
IF: intrinsic factor
MeCbl: methylcobalamin
MMA: methylmalonic acid
MMACHC: methylmalonic aciduria and homocystinuria type C protein
MM-CoA: methylmalonyl-CoA
MSR: methionine synthase reductase
TC: transcobalamin

## Supporting Information

As a service to our authors and readers, this journal provides supporting information supplied by the authors. Such materials are peer reviewed and may be re-organized for online delivery, but are not copy-edited or typeset. Technical support issues arising from supporting information (other than missing files) should be addressed to the authors.

Table 1. Selected studies showing the effectiveness and clinical or metabolic outcome after treatment with CNCbl or OHCbl. Table 2. Selected studies using MeCbl.
