# Supplementary material for: Cobalamin coenzyme forms are not likely to be superior to cyano- and hydroxyl-cobalamin in prevention or treatment of cobalamin deficiency
Source: Mol Nutr Food Res. 2015 May 12;59(7):1364–72. doi: 10.1002/mnfr.201500019 (PMC4692085; doi:10.1002/mnfr.201500019)
Supplement: Supplementary file 1 [file mnfr0059-1364-sd1.doc]

| **Table 1.** Selected studies showing the effectiveness and clinical or metabolic outcome after treatment with CNCbl or OHCbl | | | | |
| --- | --- | --- | --- | --- |
| **Study** | **Participants / experiments** | **Treatment** | **Outcome** | **Results** |
| Torsvik I, et al., [1] | Infants < 8 m with feeding difficulties, subtle neurological symptoms, delayed psychomotor development | N= 42 received 400 µg single dose of OHCbl, n=37 received sham i.m. | Evaluation 1 month later | Cbl repletion and improved neurological symptoms (motor function and regurgitations) |
| [Dhonukshe-Rutten RA](http://www.ncbi.nlm.nih.gov/pubmed?term=Dhonukshe-Rutten RA%5BAuthor%5D&cauthor=true&cauthor_uid=16155269), et al., *[2]* | Plasma B12 100- 300 pmol/L. MMA ≥ 0.30 µmol/L, 112 elderly persons | Oral CNCbl (1mg) (capsule or fortified milk) or placebo | Treatment for 12-wk, plasma B12, Hcy, MMA | Compared to the placebo, mean B12 increased by 250-280 pmol/L, MMA decreased by 0.18-0.19 µmol/L; Hcy decreased by 1.8-4.0 µmol/L. Fortified-milk and capsule are similar (*P=* 0.40). |
| Favrat B et al., [3] | 50 patients (mean age 70 y, range 31-76y) with serum B12 125-200 pmol/L | Randomized to oral CNCbl (1mg/d, N = 26) or placebo (N = 24) | Treatment for 4 weeks. The primary outcome changes in serum B12, Hcy, MMA | Lowered serum MMA by 0.13 μmol/l, increased B12 by 102 pmol/L, no significant change in Hcy (baseline mean Hcy was 18.3 µmol/L) |
| Hill MH, et al., [4] | 100 Elderly with plasma B12 <250 pmol/L and uMMA >1.5 mmol MMA/mmol creatinine. | A double-blind, placebo-controlled, randomized study using (500µg/d, 100 µg/d, and 10 µg/d CNCbl) | Intervention for 8-wk. Plasma B12, holoTC, MMA, uMMA | Increase B12 and holoTC were achieved at doses as low as 10 µg/d. Ex-smokers required 500 µg/d achieve plasma MMA lowering compared to that in non-smokers receiving 100 µg/d. |
| *Eussen S, et al., [5]* | 120 elderly with B12 level of 100 to 300 pmol/L and MMA ≥ 0.26 μmol/L. | Randomized, parallel group,double-blind, dose-finding trial. Daily oral doses of 2.5,100, 250, 500, and 1000 μg of CNCbl | Treatment for 16 weeks. Outcome to determine thelowest oral dose of CNCbl needed to normalize MMA (lowering MMA by 80-90%) | 2.5, 100, 250, 500 and 1000 µg caused 16%, 16%, 23%, 33%, and 33%, reduction in MMA. Maximal reduction of MMA was estimated to be achieved using daily doses of 647 to 1032 μg of CNCbl for 16 week. The effects were already almost maximal after 8 weeks (using 1mg/d: MMA lowered by 0.26, Hcy by 5.1 µmol/L). |
| Duggan C, et al., [6] | 183 x2 pregnant Indian women | 50 µg/d CNCbl or placebo oral, from 14 week gestation to 6 weeks post-partum | B12 status in infants and mothers | In B12 supplemented women, plasma B12 was higher during pregnancy and in milk post-partum. Maternal MMA and Hcy did not differ from the placebo group during pregnancy. Infants born to supplemented mothers had higher B12, lower MMA and lower Hcy compared to placebo. |
| Torsvik I, et al., [1] | N= 79 infants > 8 m with neurologic symptoms, and plasma tHcy 6.5-18.0 µmol/L. | Double-blind, randomized controlled either 400 µg OHCbl / i.m. (n = 42) or a sham injection (n = 37). | Changes in B12 markers and neurological symptoms | Plasma tHcy decreased by  54% (8.7 to 4.04 µmol/L), and MMA decreased by 84% (from 0.85 to 0.14 µmol/L) with marked effects on improving the neurological signs |
| Manssor, et al.,[7] | 14 patients with severe B12 deficiency (mean MMA= 5.8 µmol/L, Hcy=55 µmol/L) | 21 days with 1mg CNCbl at day 1,3, 7, 14, and 21/ i.m | Lowering B12-responsive metabolites | The first 1mg CNCbl i.m caused a significant reduction in MMA (5.8 to 4.6) and Hcy (55 to 41 µmol/L)- this effect continued over the time. |
| Shahab-Ferdows,S et al., [8] | Women from Mexico, aged 20–59 y, divided into deficient, marginal, and adequate status groups (serum B12, 75–148, 149–220, and >220 pmol/L, respectively). | Single dose of 1 mg CNCbl i.m; then 500 μg/d orally for 3 months, n = 70) or placebo (n = 62) | Serum B12, holoTC, MMA and tHcy | MMA lowered by 0.11 in the deficiency group, 0.04 in the adequate group; Hcy was lowered by 3 vs. 1 µmol/L. |
| Adams JF, et al., [9] | Patients with malabsorption or pernicious anemia | Injecting 54 µg-30 mg using Co^58^CNCbl | Measuring retention and excretion of the dose | At lower doses, the percentage of retention was higher. At higher doses the percentage of retention was lower, but the absolute amount retained was higher. Example: of 540 µg, 466 µg retained; of 8mg, 2.27mg retained. |

| **Table 2.** Selected studies using MeCbl. | | | | | |
| --- | --- | --- | --- | --- | --- |
| **Study** | **Country** | **Participant/model** | **Treatment** | **Outcome** | **Results** |
| Watanabe et al., [10] | Japan | Wistar rats | 500 µg/kg vs. 50µg/kg MeCbl | Peripheral nerve regeneration after experimental acrylamide neuropathy | Tibial nerve potential after supramaximal stimulating current was measured. High dose MeCbl was associated with a faster recovery. Animal serum and CSF B12 were > 10 fold higher under the high dose compared to the low does of MeCbl. |
| Yamazaki et al., [11] | Japan | Male gracile axonal dystrophy mutant mice model | 1 mg/kg oral  MeCbl from 40^th^ day after birth for 25 days, saline as a control | Nerve regeneration | Nerve regeneration was improved after B12: terminal perimeter is higher, terminal area is lower under MeCbl |
| Okamato et al., [12] | Japan | C2C12 cell cultures | 0,10,50,100 µM MeCbl in the culture medium | Proliferation, migration, and apoptosis | MeCbl promotes the proliferation and  migration of C2C12 myoblasts via the Erk1/2 signaling pathway, inhibits C2C12 cell apoptosis. No comparison with other forms. |
| Akaike et al., [13] | Japan | Cultured cortical neurons incubated with glutamate | Incubation with SAM or MeCbl | Cell viability | SAM and MeCbl were able to prevent glutamate neurotoxicity. No comparison with other Cbl forms. Study conclusion: the effect of MeCbl is mediated by SAM |
| Taniguchi et al.,[14] | Japan | 10 patients with diabetic neuropathy (DM since 3-13 years, 8 treated with insulin) | MeCbl 2.5 mg intraecally each month (total 2-8 injections). | Clinical outcome | Relief of heaviness in the legs, and the return of sensation within 1 week and lasted for several months. No control group. The authors hypothesized that the dramatic effect to be related to a fast effect, and the long lasting effect of relief to be related to the role of Cbl in methionine metabolism. |
| Kuwabara et al.,[15] | Japan | 10 patients on haemodialyses for 2-20years who have polyneuropathy were included. 4/10 had diabetes related renal failure. All had sensory dominant polyneuropathy | 500 µg MeCbl, i.v. three times per week for 6 months after each hemodialysis, the study was open non-controlled | Neurological disability score for grip strength, grade of pain, and paresthesia. Nerve conduction studies were also done on the median, ulnar, tibial, and sural nerves | The ulnar motor and median sensory nerve conduction velocities were significantly increased. Neuropathic pain grading was lower after 6 months. No comparison with other forms. |
| [Koyama K](http://www.ncbi.nlm.nih.gov/pubmed?term=Koyama K%5BAuthor%5D&cauthor=true&cauthor_uid=20430500), et al., [16] | Japan | 40 ESRD patients randomly assigned to | receive folate alone (15 mg/d; n = 20) or folate (15 mg/d) and MeCbl (500 µg after each hemodialysis treatment 3/ weekly for 3 weeks; n = 20 | Lowering Hcy | Co-administration of intravenous MeCbl and oral folate in hemodialysis patients normalized hyperhomocysteinemia. No comparison with other Cbl forms, combined treatment with folate. |
| Koyama K, et al., [17] | Japan | 21 patients with HD | MeCbl 500 mg intravenously (with folic acid) after each HD for 3 weeks | Lowering Hcy | Lowering fasting Hcy by 12-13 µmol/L that is 60% of baseline values when using a combination of folic acid and MeCbl. Lowered post methionine load Hcy by ca. 50% compared with the group that received only folic acid. Comparable to our results on CNCbl [18]. |
| Okada K, et al., [19] | Japan | Rats and rat cultured neurons | Cultured neurons incubated with Cbl forms for 72 hrs, rat sciatic nerve injury model in 8-wek rats treated with MeCbl for 12 weeks. | Axonal length, total neurite length, apoptosis in vitro and sensory functions in vivo | MeCbl caused a stronger reduction in apoptotic rate, enhancement in axonal length compared with (CN, OH, Ado in this order). However, the incubation was only 72 hrs. The improvement in-vivo after MeCbl (compared with PBS saline) was evident from 4-8 weeks upward. |
| Jalaludin MA, et al., [20] | Malaysia | N=60 patients with Bell´s Palsy | MeCbl (500 µg i.m. 3 /week for 8 weeks); MeCbl plus steroid; steroid | Time required for recovery; facial nerve scores, severity of illness | Better outcome on MeCbl or MeCbl plus steroid compared to steroid alone. No comparison with other Cbl forms. |
| Ide H, et al., [21] | Japan | 11 patients with diabetes neuropathy (7 M + 4F), mean age 53 yrs, diabetes for a mean of 8.7 yrs. | MeCbl (intrathecal 2.5 mg, repeated 1-3 times at 1 month intervals), 5 patients received also 1.5 mg/d MeCbl orally for 1 month | Relief of symptoms and increase nerve conduction velocity | In all patients, relief in heaviness in the legs and return of sensation less than 1 week after injection. No change in nerve conduction velocity. No control group or comparison with other Cbl forms. |
| Kikuchi et al., [22] | Japan | Glutamate induced neurotoxicity in rat retinal cell culture | Incubation with SAM or MeCbl | Cell viability | SAM and MeCbl were able to prevent glutamate neurotoxicity. No comparison with other forms. Authors conclusion: the effect of MeCbl is mediated by SAM. |
| Araki A, et al., [23] | Japan | NIDDM n=52 with macroangiopathy, n=84 without macroangiopathy, n=57 non-diabetics; age 65 y | 10 patients were treated with 1mg MeCbl i.m, daily for 3 weeks | Hcy was elevated in patients with macroangiopathy versus the other 2 groups (mean 10.8, 8.3, 7.5 µmol/L). | MeCbl lowered Hcy treated diabetics from 14.7 to 10.2 µmol/L. No control group. |

Reference List

[1]. Torsvik, I., Ueland, P. M., Markestad, T., Bjorke-Monsen, A. L. Cobalamin supplementation improves motor development and regurgitations in infants: results from a randomized intervention study. Am. J Clin Nutr. 2013, 98, 1233-1240.

[2]. Dhonukshe-Rutten, R. A., van, Z. M., de Groot, L. C., Eussen, S. J. et al. Effect of supplementation with cobalamin carried either by a milk product or a capsule in mildly cobalamin-deficient elderly Dutch persons. Am. J Clin Nutr. 2005, 82, 568-574.

[3]. Favrat, B., Vaucher, P., Herzig, L., Burnand, B. et al. Oral vitamin B12 for patients suspected of subtle cobalamin deficiency: a multicentre pragmatic randomised controlled trial. BMC. Fam. Pract. 2011, 12, 2.

[4]. Hill, M. H., Flatley, J. E., Barker, M. E., Garner, C. M. et al. A vitamin B-12 supplement of 500 mug/d for eight weeks does not normalize urinary methylmalonic acid or other biomarkers of vitamin B-12 status in elderly people with moderately poor vitamin B-12 status. J Nutr. 2013, 143, 142-147.

[5]. Eussen, S. J., de Groot, L. C., Clarke, R., Schneede, J. et al. Oral cyanocobalamin supplementation in older people with vitamin B12 deficiency: a dose-finding trial. Arch. Intern. Med. 2005, 165, 1167-1172.

[6]. Duggan, C., Srinivasan, K., Thomas, T., Samuel, T. et al. Vitamin B-12 supplementation during pregnancy and early lactation increases maternal, breast milk, and infant measures of vitamin B-12 status. J Nutr. 2014, 144, 758-764.

[7]. Mansoor, M. A., Stea, T. H., Schneede, J., Reine, A. Early biochemical and hematological response to intramuscular cyanocobalamin therapy in vitamin B(12)-deficient patients. Ann. Nutr. Metab. 2013, 62, 347-353.

[8]. Shahab-Ferdows, S., Anaya-Loyola, M. A., Vergara-Castaneda, H., Rosado, J. L. et al. Vitamin B-12 supplementation of rural Mexican women changes biochemical vitamin B-12 status indicators but does not affect hematology or a bone turnover marker. J Nutr. 2012, 142, 1881-1887.

[9]. Adams JF. The urinary excretion and retention of cyaocobalamin by subjects given repeated parenteral doses. J Clin Pathol. 1964, 17, 31-38.

[10]. Watanabe, T., Kaji, R., Oka, N., Bara, W., Kimura, J. Ultra-high dose methylcobalamin promotes nerve regeneration in experimental acrylamide neuropathy. J Neurol. Sci. 1994, 122, 140-143.

[11]. Yamazaki, K., Oda, K., Endo, C., Kikuchi, T., Wakabayashi, T. Methylcobalamin (methyl-B12) promotes regeneration of motor nerve terminals degenerating in anterior gracile muscle of gracile axonal dystrophy (GAD) mutant mouse. Neurosci. Lett. 1994, 170, 195-197.

[12]. Okamoto, M., Tanaka, H., Okada, K., Kuroda, Y. et al. Methylcobalamin promotes proliferation and migration and inhibits apoptosis of C2C12 cells via the Erk1/2 signaling pathway. Biochem. Biophys. Res Commun. 2014, 443, 871-875.

[13]. Akaike, A., Tamura, Y., Sato, Y., Yokota, T. Protective effects of a vitamin B12 analog, methylcobalamin, against glutamate cytotoxicity in cultured cortical neurons. Eur J Pharmacol. 1993, 241, 1-6.

[14]. Taniguchi, H., Ejiri, K., Baba, S. Improvement of autonomic neuropathy after mecobalamin treatment in uremic patients on hemodialysis. Clin Ther. 1987, 9, 607-614.

[15]. Kuwabara, S., Nakazawa, R., Azuma, N., Suzuki, M. et al. Intravenous methylcobalamin treatment for uremic and diabetic neuropathy in chronic hemodialysis patients. Intern Med. 1999, 38, 472-475.

[16]. Koyama, K., Ito, A., Yamamoto, J., Nishio, T. et al. Randomized controlled trial of the effect of short-term coadministration of methylcobalamin and folate on serum ADMA concentration in patients receiving long-term hemodialysis. Am. J Kidney Dis. 2010, 55, 1069-1078.

[17]. Koyama, K., Usami, T., Takeuchi, O., Morozumi, K., Kimura, G. Efficacy of methylcobalamin on lowering total homocysteine plasma concentrations in haemodialysis patients receiving high-dose folic acid supplementation. Nephrol. Dial. Transplant. 2002, 17, 916-922.

[18]. Obeid, R., Kuhlmann, M. K., Kohler, H., Herrmann, W. Response of homocysteine, cystathionine, and methylmalonic acid to vitamin treatment in dialysis patients. Clin Chem. 2005, 51, 196-201.

[19]. Okada, K., Tanaka, H., Temporin, K., Okamoto, M. et al. Methylcobalamin increases Erk1/2 and Akt activities through the methylation cycle and promotes nerve regeneration in a rat sciatic nerve injury model. Exp. Neurol. 2010, 222, 191-203.

[20]. Jalaludin, M. A. Methylcobalamin treatment of Bell's palsy. Methods Find. Exp. Clin Pharmacol. 1995, 17, 539-544.

[21]. Ide, H., Fujiya, S., Asanuma, Y., Tsuji, M. et al. Clinical usefulness of intrathecal injection of methylcobalamin in patients with diabetic neuropathy. Clin Ther. 1987, 9, 183-192.

[22]. Kikuchi, M., Kashii, S., Honda, Y., Tamura, Y. et al. Protective effects of methylcobalamin, a vitamin B12 analog, against glutamate-induced neurotoxicity in retinal cell culture. Invest Ophthalmol. Vis. Sci. 1997, 38, 848-854.

[23]. Araki, A., Sako, Y., Ito, H. Plasma homocysteine concentrations in Japanese patients with non-insulin-dependent diabetes mellitus: effect of parenteral methylcobalamin treatment. Atherosclerosis. 1993, 103, 149-157.
